# Interleukin 12 shows a better curative effect on lung cancer than paclitaxel and cisplatin doublet chemotherapy

**DOI:** 10.1186/s12885-016-2701-7

**Published:** 2016-08-22

**Authors:** Ting Yue, Xiaodong Zheng, Yaling Dou, Xiaohu Zheng, Rui Sun, Zhigang Tian, Haiming Wei

**Affiliations:** 1 Institute of Immunology, School of Life Sciences, University of Science and Technology of China, Hefei, Anhui China; 2 Hefei National Laboratory for Physical Sciences at Microscale, University of Science and Technology of China, Hefei, Anhui China

**Keywords:** Interleukin-12, Lung cancer, Chemotherapy, Natural Killer cells, Interferon-γ, Angiogenic

## Abstract

**Background:**

Interleukin 12 (IL-12) is a cytokine that has been reported to exhibit potent tumoricidal effects in animal tumor models. A combined approach using Paclitaxel and platinum-based doublet chemotherapy is the most commonly used backbone regimen for treating lung cancer. Despite numerous studies regarding the anti-tumor effects of IL-12 and the widespread use of conventional chemotherapy, few direct comparisons of IL-12 and conventional chemotherapy in the treatment of lung cancer have been performed.

**Methods:**

We compared IL-12 to paclitaxel and cisplatin doublet chemotherapy in terms of efficacy against lung cancer in mouse models. The antitumor effect was measured by survival assays, histological analyses and imaging analyses. The cytokine levels were assessed using enzyme linked immunosorbent assay (ELISA) and flow cytometry (FACS). The spleen sizes were measured. CD31, CD105 and Vascular endothelial growth factor receptor 3 (VEGFR3) were analyzed using immunofluorescence. Matrix metalloprotein-9 (MMP-9) and cadherin 1 (CDH1) transcript levels were measured by quantitative PCR. Tumor cells apoptosis were examined by Tunel assay.

**Results:**

The results showed that IL-12 treatment inhibited lung tumor growth, resulting in the long-term survival of lung cancer-bearing mice. Further examination revealed that IL-12 rapidly activated NK cells to secrete IFN-γ, resulting in the inhibition of tumor angiogenesis. In contrast, paclitaxel and cisplatin doublet chemotherapy did not show the expected efficacy in orthotopic lung cancer models; the IFN-γ levels were not increased after this treatment, and the number of peripheral lymphocytes was reduced.

**Conclusion:**

Together, these animal model data indicate that IL-12 shows a better curative effect than PTX + CDDP doublet chemotherapy.

**Electronic supplementary material:**

The online version of this article (doi:10.1186/s12885-016-2701-7) contains supplementary material, which is available to authorized users.

## Background

Lung cancer is one of the most commonly diagnosed cancers and the leading cause of cancer deaths globally, causing more than 1.4 million deaths annually [1, 2]. Paclitaxel (PTX) and cisplatin (CDDP) doublet chemotherapy is one of the first-line treatments for patients with non-small cell lung cancer (NSCLC) and small cell lung cancer (SCLC) [3–5]. Although this chemotherapeutic approach can be effective, the survival rates remain low [3, 6, 7]. Therefore, effective therapeutic approaches such as immunotherapies are urgently needed.

Interleukin 12 (IL-12) is a pro-inflammatory cytokine that was originally identified as a natural killer (NK) cell stimulatory factor (NKSF) and a cytotoxic lymphocyte maturation factor [8, 9]. Physiologically, IL-12 has been implicated in the stimulation of NK and T cell proliferation, the enhancement of NK and CD8^+^ T cell cytolytic activity and the induction of cytokine production, particularly IFN-γ [10, 11]. Furthermore, IL-12 promotes the differentiation of T helper 1 (T_H_1) cells, thereby bridging innate and adaptive immunity [12, 13]. Mice lacking the IL-12 subunit p35 showed the early appearance and development of a greater number of papilloma compared with wild-type (WT) mice. Accordingly, the growth of B16 melanomas is faster in mice that are deficient in the IL-12 receptor chain IL-12Rβ2 compared to WT mice [14, 15]. Exogenously administered IL-12 exhibits impressive anti-tumor effects in different murine tumor models such as sarcoma, melanoma, lung carcinoma and breast carcinoma [16–18]. Although IL-12 has certain side effects, its curative effect is significant. The results from IL-12 phase I/II trials in patients with B-cell lymphoma or Kaposi sarcoma offer great clinical prospects [19, 20]. Furthermore, recent studies have shown that IL-12 plus IL-18 can restore intratumoral NK cell functions in MHC (major histocompatibility complex) class I-deficient tumors [21, 22].

PTX is a cytoskeletal drug that targets tubulin. By stabilizing microtubules, PTX arrests the cell cycle in the G0/G1 and G2/M phases and induces cancer cell death [23]. CDDP is a platinum coordination compound that has the ability to crosslink the purine bases in DNA, causing DNA damage and subsequently inducing apoptosis in cancer cells [24]. A combined approach using PTX and platinum-based doublet chemotherapy is the most commonly used backbone regimen for treating NSCLC and SCLC [3–5]. The effective induction of chemotherapy has been shown to be beneficial for the survival of some lung cancer patients. However, a few retrospective studies have shown that the benefits of chemotherapeutic agents were small for patients with stable disease (SD), and many cases have shown the failure of conventional chemotherapy in treating advanced NSCLC [25, 26].

In the present study, we generated two different orthotopic lung cancer models and showed that IL-12 administration could suppress tumor growth in these models, leading to long-term survival compared with the controls. In contrast, PTX + CDDP doublet chemotherapy did not improve survival compared with the control in the lung cancer models. To explore the mechanisms underlying this phenomenon, we examined IFN-γ, which is an important anti-tumor effector molecule, and found that the IFN-γ levels were significantly increased after IL-12 administration. Further examination revealed that NK cells were quickly activated and secreted IFN-γ a few hours after IL-12 administration. In contrast, the IFN-γ levels were not increased after PTX and CDDP doublet chemotherapy, and the number of peripheral lymphocytes was reduced. Furthermore, the anti-tumor activity of IL-12 was found greatly reduced in both NK cell-deficient mice and IFN-γ-deficient mice, suggesting that NK cells and IFN-γ are the primary factors that mediate the anti-tumor effects of IL-12 in lung cancer models. Moreover, we observed that IL-12 inhibited tumor angiogenesis via an IFN-γ-dependent mechanism. These results suggest that IL-12 showed a greater efficacy than PTX + CDDP doublet chemotherapy and may thus provide an effective strategy for treating lung cancer.

## Methods

### Mice and cell lines

Female C57BL/6 and BALB/c mice at 7–8 weeks of age were purchased from the Shanghai Laboratory Animal Center (Shanghai, China). Female IFN-γ^−/−^ mice on a C57BL/6 background were a generous gift from Dr. Shaobo Su (Shantou University Medical College, Shantou, China). Female CD4^−/−^ and CD8^−/−^ mice, all on a C57BL/6 background, were obtained from Prof. Zhexiong Lian (University of Science and Technology of China, Hefei, China). Female Nuclear factor interleukin-3 (Nfil3)^−/−^ mice (without NK cells) on a C57BL/6 background were a kind gift from Prof. Tak Wah Mak (University of Toronto, Toronto, Canada). All mice were maintained under specific pathogen-free conditions, and all animal experimental protocols were approved by the Ethics Committee of Animal Experiments of the University of Science and Technology of China (Approval Number: USTCACUC1201051).

LLC cells were obtained from the Chinese Academy of Sciences Cell Bank (Shanghai, China) and cultured at 37 °C with 5 % CO_2_ in DMEM supplemented with 10 % FCS. CT26 cells (a generous gift from Prof. Xuetao Cao, Zhejiang University School of Medicine, Hangzhou, China) were cultured at 37 °C with 5 % CO_2_ in RPMI 1640 supplemented with 10 % FCS.

### Tumor generation and treatment

C57BL/6 mice were injected intrapleurally with 5 × 10^5^ LLC cells in 0.1 ml of PBS to generate an orthotopic lung cancer model. The tumor-bearing mice were divided randomly into four groups for imaging and histological analyses or into five groups for survival analysis. These groups were administered saline, single doses of PTX (5 mg/kg of body weight, Cat. No. H20063662, Beijing HWELLS Co., Ltd.) and CDDP (5 mg/kg of body weight, Cat. No. H20063662, Nanjing Pharmaceutical Co., Ltd.), three doses of PTX + CDDP (5 mg/kg of body weight), and a combined treatment with recombinant murine IL-12 (rmIL-12; 12 μg/kg of body weight, Cat. No. 210–12, PeproTech). PTX+ CDDP were injected intravenously 5 days after tumor inoculation. For the survival analysis, the tumor-bearing mice were administered three doses of PTX + CDDP once a week. Eight days after tumor inoculation, three doses of IL-12 were injected subcutaneously into the forelimb root of the mice once every other day. Two days later, this treatment regimen was repeated one time.

To establish a lung metastasis model, BALB/c mice received intravenous tail vein injections of 4 × 10^4^ CT26 cells in 0.2 ml of PBS. With the exception of the group that received three doses of PTX+ CDDP, the groups and treatments were the same as those described above.

To generate lung cancer models, Nfil3^−/−^, CD4^−/−^, CD8^−/−^ and IFN-γ^−/−^ mice were injected with LLC cells as described above. PTX and CDDP were not administered to these models, and the IL-12 treatment was administered as described above.

### Bioluminescence imaging and analysis

The plasmid vector pcDNA3.1 (Cat. No. V790-20, Invitrogen) was engineered and modified to express luciferase (pcDNA3.0-Luc). LLC and CT26 cells were transfected with pcDNA3.0-Luc, and the clones with strong bioluminescence signals (150 μg/ml luciferin, Cat. No. LUCK-1G, Gold Biotechnology) were expanded and injected into mice to generate a lung cancer model. After intravenous tail vein injection of luciferin (150 μg/g body weight), the mice were anesthetized with isoflurane and placed into an IVIS imaging chamber (Caliper Life Sciences, USA). To analyze the bioluminescence images, bioluminescent signal emission regions in whole mice or lung tissue samples were designated, and the total photon flux per second was quantified and analyzed using the Living Image software (Xenogen, USA).

### Flow cytometric analysis

The spleens of mice were harvested and weighed. Spleen size was calculated as (width × length × thickness)/2. To harvest splenocytes, spleens were cut into small pieces and passed through 200-gauge mesh. Splenocytes were harvested after RBC lysis and washing. For the intracellular cytokine analysis of IFN-γ, the splenocytes (1 × 10^6^ cell/ml) were incubated for 4 h with PMA (30 ng/ml, Cat. No. P1585, Sigma-Aldrich), ionomycin (1 μg/ml, Cat. No. 407952, Merck) and monensin (5 μg/ml; Cat. No. 46468, Sigma-Aldrich). Subsequently, the splenocytes were washed and blocked to eliminate non-specific binding using anti-CD16/32 (2.4G2; BD Biosciences). FITC-anti-NK1.1 and APC-anti-CD3 (BD Biosciences, USA) were used to stain the extracellular markers. Then, the cells were fixed, permeabilized, and stained with PE-anti-IFN-γ. The stained cells were analyzed using a FACSCalibur flow cytometry system (BD Biosciences), and the data were analyzed using the FlowJo 7.6 software (Treestar, USA).

### ELISA for cytokine detection

Mouse serum was collected 1 day after treatment. The levels of IFN-γ were measured using an IFN-γ ELISA kit (Cat. No. DKW12-2000-096, Dakewe Biotech Company) according to the manufacturer’s instructions.

### Hematoxylin and eosin staining

For the histological analysis, the lung and tumor tissue sections were fixed in 10 % phosphate-buffered formalin (pH 7.2) and embedded in paraffin. The tissue sections (6-μm-thick) were affixed to the sides, deparaffinized, stained with H&E and examined under a light microscope (Zeiss, Germany).

### Immunofluorescence staining

Lung tumor samples were embedded in optimal cutting temperature compound. Cryosections (9 μm thick) were air-dried, fixed for 10 min at room temperature using a 1:1 mixture of acetone and methanol and subsequently blocked to eliminate non-specific binding using 0.5 % bovine serum albumin (BSA) in PBS for 1 h. The cryosections were incubated with PE-anti-CD31 (BD Biosciences), rabbit anti-VEGFR3 (Cat. No. ab27278, Abcam), PE-CD105 (BD Biosciences), PE-CD3 and APC-F4/80(BD Biosciences) for 12 h at 4 °C. After the cryosections were washed with PBS, they were incubated with FITC-goat anti-rabbit IgG (Cat. No. sc-2012, Santa Cruz Biotechnology) for 2 h at 37 °C and subsequently washed with PBS. The slides were stained with DAPI (Cat. No. sc-3598, Santa Cruz Biotechnology) for 2 min. Then, the slides were washed in PBS, and coverslips were mounted onto the slides using anti-fade reagent (Cat. No. P36930, Life Technologies). The images were acquired using a Zeiss LSM 710 confocal microscope (Zeiss, Germany). The expression regions of CD31, VEGFR3 and CD105 were quantified and analyzed using the Image-Pro Plus software (Media Cybernetics, USA).

### Quantitative RT-PCR analysis

Total RNA from tumor tissues was isolated using TRIzol reagent (Catalog No. 51–0700, Invitrogen, Camarillo, CA, USA). RNA was then reverse transcribed using Moloney murine leukemia virus reverse transcriptase (Catalog No. 51–0700, Invitrogen). Quantitative PCR analysis was performed according to the instructions using a SYBR Premix Ex Taq (Takara Japan). For analysis, the expression of target genes was normalized to the β-actin. All primers were synthesized by Sangon Biotech (China). The primers used to amplify β-actin were β-actin-F (5′- TTG CCG ACA GGA TGC AGA A-3′) and β-actin-R (5′- GCC GAT CCA CAC GGA GTA CTT -3′). The primers used to amplify MMP-9 were MMP-9-F (5′- GCA GAG GCA TAC TTG TAC CG -3′) and MMP-9-R (5′- TGA TGT TAT GAT GGT CCC ACT TG -3′). The primers used to amplify CDH1 were CDH1-F (5′- CAG GTC TCC TCA TGG CTT TGC -3′) and CDH1-R (5′- CTT CCG AAA AGA AGG CTG TCC -3′). The primers used to amplify IFN-γ were IFN-γ-F (5′- AAC GCT ACA CAC TGC ATC T -3′) and IFN-γ-R (5′- GAG CTC ATT GAA TGC TTG G -3′).

### Tunel analysis

LLC tumor cells were first co-cultured with PTX + CDDP (same concentration used in mice) for 12 h. Then, these cells were examined by micro-imaging and Tunel. Cryosections of tumor tissues were also examined by Tunel. Tunel analysis was performed according to the manufacturer’s instructions using a One Step TUNEL Apoptosis Assay Kit (Beyotime Biotechnology China). The images were acquired using a OLYMPUS IX81 inverted microscope (OLYMPUS, Japan) and a Zeiss LSM 710 confocal microscope (Zeiss, Germany).

### Statistical analysis

The data are shown as the means ± standard error of the mean (SEM). Significant differences between more than two groups were determined using ANOVA. Comparisons between two groups were performed using two-tailed unpaired Student’s *t*-tests (*, *P* < 0.05; **, *P* < 0.01). Survival curves were estimated using the Kaplan-Meier method, and differences between the groups were determined using the log-rank test at a minimal *P* value.

## Results

### IL-12 shows more efficacy than PTX + CDDP doublet chemotherapy in orthotopic lung cancer models

**Fig. 1:**
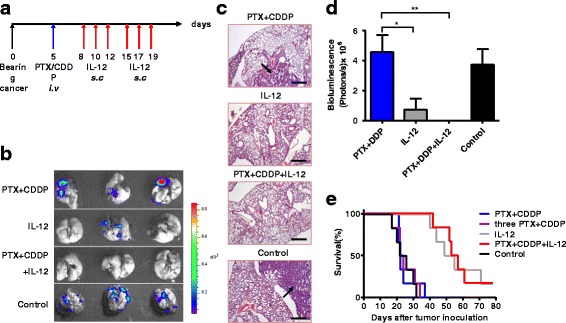
Comparison of the efficacy of IL-12 and PTX + CDDP doublet chemotherapy on LLC tumor-bearing mice. **a** Treatment regimen in orthotopic lung cancer models. Tumor-bearing mice were treated with PTX + CDDP + IL-12, IL-12, PTX + CDDP, saline (as shown). **b** Imaging of lung tumor tissues. Fewer lung tumor nodes were detected in IL-12 and PTX + CDDP + IL-12 treatment groups as compared to PTX + CDDP and control groups. **c** Pathological examination also showed that fewer lung tumor nodes were detected in IL-12 and PTX + CDDP + IL-12 treatment groups as compared to PTX + CDDP and control groups. The arrows indicate the areas of the tumor nodes (magnification × 100; scale bar, 200 μm). **d** The Imaging results were quantified and analyzed. Bioluminescence (photos/s) indicated that, after IL-12 or PTX + CDDP + IL-12 treatment, living tumor cells were markedly decreased compared with PTX + CDDP and control groups (*n* = 3; means ± SEM, *P < 0.05). **e** The survival (Kaplan-Meier) curves of the treated animals are shown; Prolonged survival was observed in IL-12 or PTX + CDDP + IL-12 group as compared to PTX + CDDP or control group (log-rank test, *P* < 0.01)

**Fig. 2:**
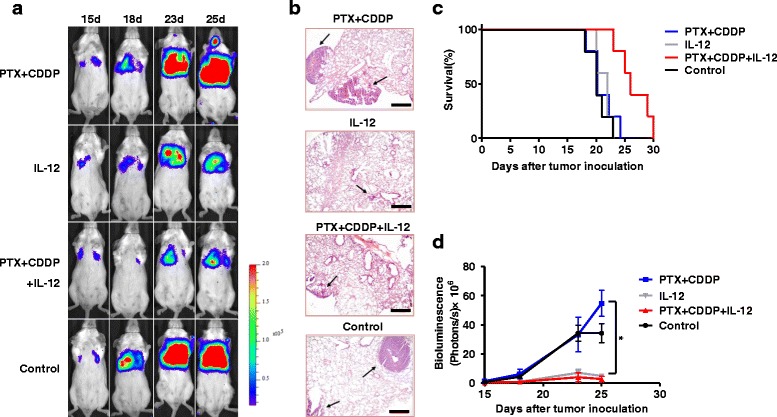
Comparison of the efficacy of IL-12 and PTX + CDDP doublet chemotherapy on CT26 tumor-bearing mice. **a** Whole-body imaging of tumor-bearing mice on different dates. Expansion of the tumors of IL-12 and PTX + CDDP + IL-12 groups was less than that of PTX + CDDP or control groups. **b** Pathological examination also showed that fewer lung tumor nodes were detected in IL-12 and PTX + CDDP + IL-12 treatment groups as compared to PTX + CDDP and control groups. The arrows indicate the areas of the tumor nodes (magnification × 100; scale bar, 200 μm). **c** The survival (Kaplan-Meier) curves of the treated animals are shown; A prolonged survival time was observed in PTX + CDDP + IL-12 group as compared to PTX + CDDP or control group (log-rank test, *P* < 0.01). **d** The imaging results were quantified and analyzed. The bioluminescence (photos/s) indicated that after IL-12 or PTX + CDDP + IL-12 treatment tumor growth was markedly slowed compared with PTX + CDDP and control groups (*n* = 3; means ± SEM, *P < 0.05)

To test the efficacy of IL-12 and PTX + CDDP doublet chemotherapy in the treatment of lung cancer, we generated two different lung cancer models. As shown in Fig. 1, after treatment with IL-12 or chemotherapy in combination with IL-12 (PTX + CDDP + IL-12), markedly fewer lung tumor nodes (the bioluminescent signal emission region, with the arrows indicating areas of the hematoxylin and eosin (H&E)-stained sections) were detected in the treatment groups compared with both the control group and the chemotherapy-treated LLC lung cancer models. We also conducted a survival assay. As shown in Fig. 1e, tumor-bearing mice treated with IL-12 or PTX + CDDP + IL-12 showed significant long-term survival compared with the control and chemotherapy treatment groups. Remarkably, the mice treated with IL-12 or PTX + CDDP + IL-12 survived more than 80 days (Fig. 1e), which suggests the anti-tumor efficacy of IL-12 as monotherapy or in combination with PTX + CDDP. Although PTX and CDDP have been used as a first-line chemotherapy treatment for lung cancer, these data showed that neither a single dose nor three doses of PTX-CDDP doublet chemotherapy were sufficient to extend the lives of tumor-bearing mice significantly or to suppress tumor growth (Fig. 1b-e). Similar results were shown in the CT26 lung metastasis model (Fig. 2a-d). In this lung cancer model, the PTX + CDDP + IL-12 treatment showed a better therapeutic effect than the PTX + CDDP treatment alone and resulted in long-term survival (Fig. 2c). We also counted the visible tumor nodes and measured the weight of tumor and lung tissues as a whole after completion of the treatments. These data revealed that the IL-12 or PTX + CDDP + IL-12 treated mice had less visible lung tumor nodes and lower lung tumor weight compared to the PTX + CDDP or control groups (Additional file 1: Figure S1). To assess whether PTX + CDDP has an effect on tumor cells, in vitro and in vivo experiments were performed. The results showed that PTX + CDDP (same concentration used in mice) treatment resulted in LLC cell apoptosis in vitro, however, no apoptosis was detected in LLC tumor tissues after PTX + CDDP treatment (Additional file 2: Figure S2). Based on these data, IL-12 showed a greater efficacy than PTX + CDDP doublet chemotherapy in the two different lung cancer models.

### IL-12 activates the immune system and rapidly stimulates NK cells to secrete IFN-γ

**Fig. 3:**
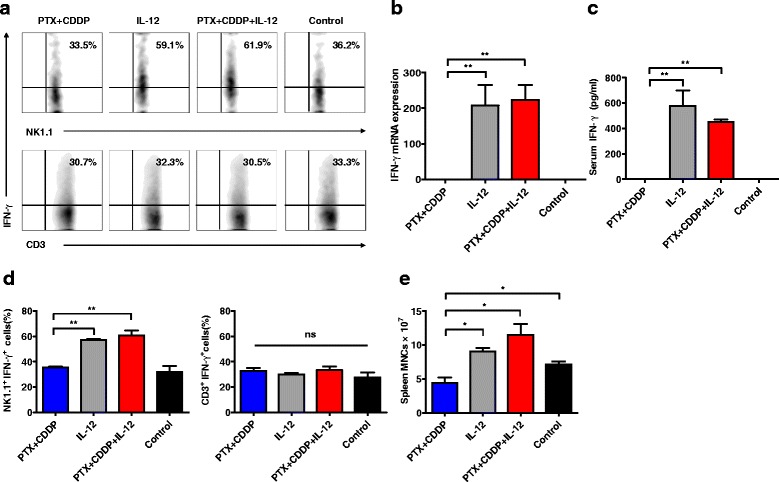
IL-12 activates the immune system and rapidly stimulates NK cells to secrete IFN-γ. **a** Flow cytometry analysis of IFN-γ-producing lymphocytes isolated from the spleens of tumor-bearing mice. The expression of IFN-γ in NK cells increased significantly after IL-12 or PTX + CDDP + IL-12 treatment as compared to PTX + CDDP treatment or control. **b** IFN-γ transcript levels in tumor tissues of different groups were measured by quantitative PCR. The IFN-γ transcript levels in IL-12 or PTX + CDDP + IL-12 groups were much higher than those in the PTX + CDDP or control groups. (*n* = 3; means ± SEM, **P < 0.01). **c** The IFN-γ serum concentration of different treatments in tumor-bearing mice. The serum IFN-γ levels in IL-12 or PTX + CDDP + IL-12 groups were much higher than those in the PTX + CDDP or control groups. (*n* = 3; means ± SEM, **P < 0.01). **d** The percentages of NK1.1^+^IFN-γ^+^ and CD3^+^IFN-γ^+^ cells in the spleens of tumor-bearing mice in different groups. The percentages of NK1.1^+^IFN-γ^+^ in IL-12 or PTX + CDDP + IL-12 groups were much higher than those in the PTX + CDDP or control groups. (*n* = 6; means ± SEM, **P < 0.01). **e** The spleen mononuclear cell numbers from tumor-bearing mice. More spleen mononuclear cells were found in the IL-12 or PTX + CDDP + IL-12 groups as compared to PTX + CDDP and control groups. Less spleen mononuclear cells were found in the PTX + CDDP group compared to the control group (*n* = 6; means ± SEM, *P < 0.05)

To investigate the effects of IL-12 and PTX + CDDP doublet chemotherapy on the immune system, tumor-bearing mice were treated with IL-12, PTX + CDDP or NS and then sacrificed. Surprisingly, we observed that the spleens of tumor-bearing mice treated with IL-12 or PTX + CDDP + IL-12 were markedly enlarged but that the spleens of tumor-bearing mice treated with PTX + CDDP alone were slightly shrunken compared with the spleens of control mice (Additional file 3: Figure S3). Subsequently, we isolated mononuclear cells from the spleens of these mice and quantified the cells. Notably, the number of mononuclear cells from the spleens of tumor-bearing mice treated with IL-12 or PTX + CDDP + IL-12 increased significantly compared with both the PTX + CDDP-treated mice and the control mice (Fig. 3e). We examined the IFN-γ transcript levels in tumor tissues of different groups by quantitative PCR. The results showed that the IFN-γ transcript levels in the tumor tissues of the IL-12 or PTX + CDDP + IL-12 groups were much higher than those in the control group that was treated with PTX + CDDP alone (Fig. 3b). We also used ELISA to measure the serum IFN-γ levels in tumor-bearing mice following treatment. As expected, the serum IFN-γ levels in the tumor-bearing mice that were treated with IL-12 or PTX + CDDP + IL-12 were much higher than were those in the control mice. By contrast, almost no IFN-γ was detected in the sera of tumor-bearing mice treated with PTX + CDDP (Fig. 3c). To further identify the immune cell subpopulation responsible for IFN-γ production, we examined the expression of intracellular IFN-γ using flow cytometry (Fig. 3a). Interestingly, IFN-γ secretion by NK cells increased markedly and rapidly following IL-12 or PTX + CDDP + IL-12 treatment. However, the NK cells showed no IFN-γ secretion after PTX + CDDP treatment compared with the control (Fig. 3d). These data suggest that IL-12 can activate NK cells rapidly to produce IFN-γ, whereas PTX + CDDP not only failed to promote IFN-γ production but also reduced the number of peripheral lymphocytes to some extent.

### NK cells and IFN-γ are essential for the anti-tumor effects of IL-12

**Fig. 4:**
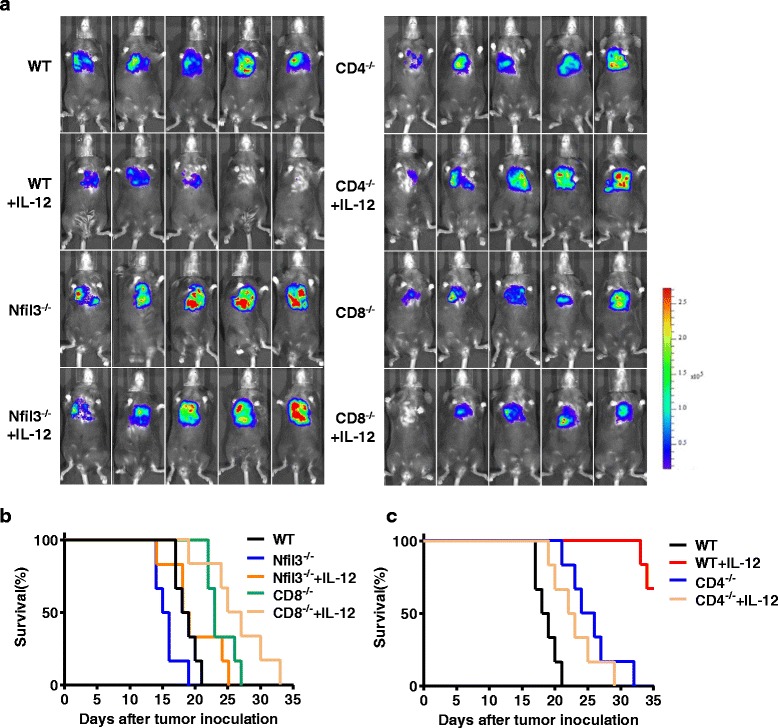
NK cells are essential for the anti-tumor effects of IL-12. **a** Whole-body imaging of WT, Nfil3^−/−^, CD4^−/−^ and CD8^−/−^ tumor-bearing mice. Tumors detected in the Nfil3^−/−^ group grew more robustly compared to the other groups whether IL-12 was administered or not. **b** The survival (Kaplan-Meier) curves of the treated animals are shown; No prolonged survival time was observed in the Nfil3^−/−^ + IL-12 group as compared to the other groups. **c** The survival (Kaplan-Meier) curves of the treated animals are shown; A prolonged survival time was observed in the WT + IL-12 group as compared to other groups (log-rank test, *P* < 0.01)

**Fig. 5:**
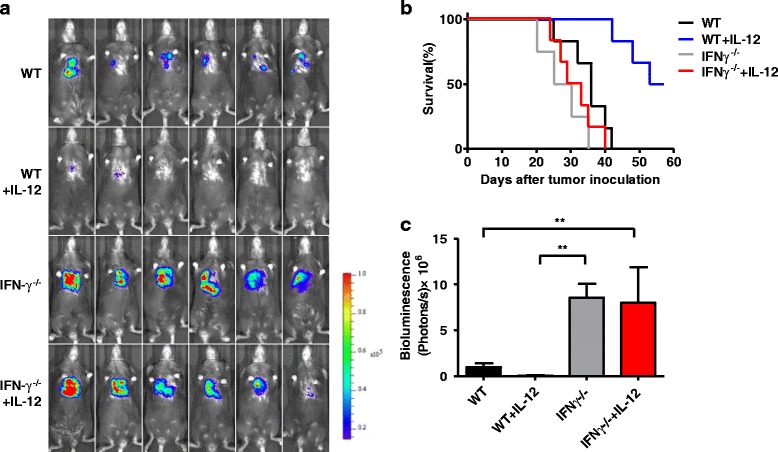
The anti-tumor effect of IL-12 is dependent on IFN-γ. **a** Whole-body imaging of WT and IFN-γ^−/−^ tumor-bearing mice. Tumors detected in the IFN-γ^−/−^ groups grew more robustly compared with WT group whether IL-12 was administered or not. **b** The survival (Kaplan-Meier) curves of the treated animals are shown. A prolonged survival time was observed in the WT + IL-12 group as compared to other groups (log-rank test, *P* < 0.01). No prolonged survival time was observed in the IFN-γ^−/−^ + IL-12 group. **c** The imaging results were quantified and analyzed. The bioluminescence (photos/s) indicated that the tumors in IFN-γ^−/−^ mice grew more robustly compared to the WT group whether IL-12 was administered or not. (*n* = 6; means ± SEM, **P < 0.01)

Although earlier results indicate that NK cells in tumor-bearing mice can be activated to produce IFN-γ following IL-12 treatment, the role of NK cells in the IL-12-mediated lung tumor suppression still needs to be investigated. To further confirm the roles of NK and IFN-γ in the anti-tumor effects of IL-12, Nfil3^−/−^ (without NK cells), CD4^−/−^, CD8^−/−^, IFN-γ^−/−^ and WT mice were injected with LLC tumor cells to establish lung cancer models. Subsequently, the tumor-bearing mice were treated with IL-12 or NS, and tumor growth was monitored via whole-body imaging (Figs. 4a and 5a). Remarkably, the tumors in Nfil3^−/−^ mice or in IFN-γ^−/−^ mice grew significantly faster than did those in the WT, CD4^−/−^ and CD8^−/−^ mice, as indicated by the bioluminescent signal emission region. Furthermore, IL-12 treatment inhibited tumor growth in the WT mice but not in the Nfil3^−/−^ and IFN-γ^−/−^ mice (Fig. 4a and 5a-c). These results were also confirmed by survival analyses, which indicated that the tumor-bearing WT mice exhibited significant long-term survival compared with the tumor-bearing Nfil3^−/−^ and IFN-γ^−/−^ mice after treatment with IL-12 (Figs. 4b and 5b). We also performed an immunofluorescence assay to evaluate T cell and macrophage infiltration in tumors. However, similar macrophage recruitment was observed in all groups, and all tumor sections exhibited a minimal infiltration of T cells (Additional file 4: Figure S4). Collectively, these data indicate that NK cells and IFN-γ play essential roles in the anti-tumor effects of IL-12 in these lung cancer models.

### IL-12 via mediation of IFN-γ suppresses tumor angiogenesis

**Fig. 6:**
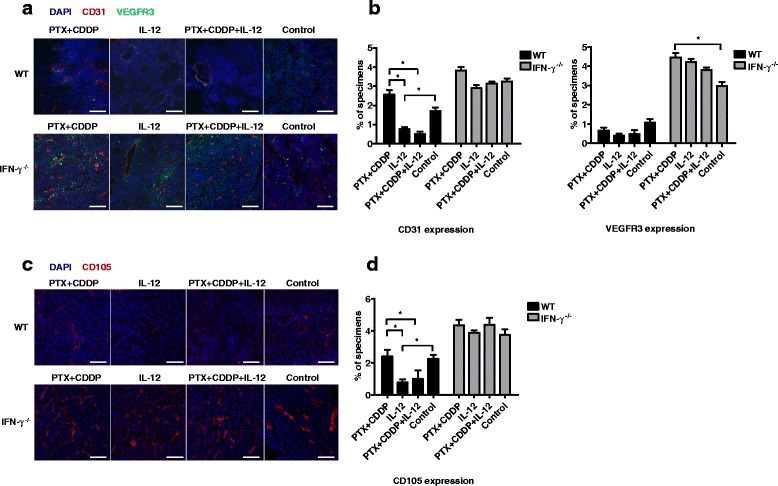
IL-12-induced IFN-γ suppresses tumor angiogenesis. **a** Immunofluorescence of tumor tissues in WT and IFN-γ^−/−^ tumor-bearing mice. CD31 and VEGFR3 expression in the lung tumor tissues of WT mice was reduced after IL-12 or PTX + CDDP + IL-12 treatment (original magnification, ×100; scale bar, 100 μm). **b** Percentage of specimens showing CD31 or VEGFR3 expression. CD31 and VEGFR3 expression in the lung tumor tissues of IFN-γ^−/−^ mice was higher than that of WT. CD31 expression in the lung tumor tissues of WT mice was reduced after IL-12 or PTX + CDDP + IL-12 treatment (*n* = 3; means ± SEM, **P < 0.01). **c** Immunofluorescence of tumor tissues in WT and IFN-γ^−/−^ tumor-bearing mice. CD105 expression in the lung tumor tissues of WT mice was reduced after IL-12 or PTX + CDDP + IL-12 treatment (original magnification, ×100; scale bar, 100 μm). **d** Percentage of specimens showing CD105 expression. CD105 expression in the lung tumor tissues of IFN-γ^−/−^ mice was higher than that of WT. CD105 expression in the lung tumor tissues of WT mice was reduced after IL-12 or PTX + CDDP + IL-12 treatment (*n* = 3; means ± SEM, **P < 0.01)

To investigate whether IL-12 via mediated IFN-γ expression suppresses tumor angiogenesis in lung cancer, IFN-γ^−/−^ and WT mice were tumor-burdened and subsequently treated with IL-12. After the mice received treatments, lung tumor tissue sections were processed, and the frozen lung tumor tissue sections were stained with anti-CD31 and anti-vascular endothelial growth factor receptor 3 (VEGFR3). Confocal immunofluorescence imaging showed that the lung tumor tissues from IFN-γ^−/−^ mice had higher levels of CD31 and VEGFR3 expression than did those from WT mice (Fig. 6a). Moreover, IL-12 or PTX + CDDP + IL-12 treatment reduced CD31 and VEGFR3 expression in the lung tumor tissues of WT mice (Fig. 6a and b). In addition, we observed the co-localization of CD31 and VEGFR3 in the lung tumor tissues of WT mice after IL-12 or PTX + CDDP + IL-12 treatment. However, IL-12 or PTX + CDDP + IL-12 treatment did not reduce CD31 and VEGFR3 expression in the lung tumor tissues of IFN-γ^−/−^ mice. In these animals, CD31 and VEGFR3 did not co-localize and showed dysregulated expression (Fig. 6a). We also examined another vascular marker CD105 and found that its expression was reduced in wildtype but not in IFN-γ^−/−^ tumor-bearing mice after IL-12 treatment (Fig. 6c and d). Reduced blood vessels may cause an increase in hypoxia that result in more invasive phenotype [27, 28]. To evaluate invasive phenotype of different groups, we examined the transcript levels of matrix metalloprotein-9 (MMP-9) and cadherin 1 (CDH1) in para-carcinoma tissue. The results showed that the MMP-9 transcript level was decreased after IL-12 treatment; however, the CDH1 transcript level did not change significantly (Additional file 5: Figure S5). These results may suggest that treatment with IL-12 does not result in a more invasive phenotype. Taken together, these data indicate that the IL-12 via mediated inhibition of tumor angiogenesis is IFN-γ dependent and may suppress tumor growth.

## Discussion

Lung cancer is the leading cause of cancer deaths worldwide [1, 2]. Although traditional lung cancer treatments such as surgery, chemotherapy and radiotherapy remain widely used, the results of such treatments are not satisfactory [7]. Thus, different therapeutic approaches including immunotherapies are being studied.

As a type of immunotherapy, IL-12 has displayed significant anti-tumor effects in a variety of animal models [29–31]. However, conventional chemotherapy remains one of the most commonly used treatments for lung cancer. Therefore, comparing the efficacy of IL-12 with conventional chemotherapies or testing the efficacy of a combination of IL-12 and chemotherapy against lung cancer is important. PTX + CDDP doublet chemotherapy is recommended for treating NSCLC and SCLC [5, 32]. However, our preliminary results showed that PTX + CDDP treatment resulted in LLC cell apoptosis in vitro, and no apoptosis was detected in LLC tumor tissues after PTX + CDDP treatment (Additional file 2: Figure S2). Here, we established two different orthotopic lung cancer models (Figs. 1 and 2). In contrast to subcutaneous tumor models, tumor nodes bud from lung tissues in orthotopic lung cancer models, thereby mimicking actual lung cancer conditions. To determine the treatment and dosage regimens of chemotherapy and IL-12, we referred to previous reports [16, 33–35] and the results of preliminary experiments.

Interestingly, IL-12 administration via axillary subcutaneous injection significantly inhibited lung tumor growth, resulting in the long-term survival of tumor-bearing mice (Figs. 1 and 2). However, despite being administered in either single or multiple doses, PTX + CDDP doublet chemotherapy did not show the expected efficacy and improvement in the survival of the lung cancer models compared with the controls (Figs. 1 and 2). Conventional chemotherapy has been thought to act via the direct killing of tumor cells [36]. However, chemotherapy also affects other systems of the body, such as the immune system [37]. Differing views exist regarding the role of conventional chemotherapy on the immune system [36–38]. Evidence has suggested that conventional chemotherapy drugs such as cyclophosphamide may induce tumor cell death and stimulate the cross-presentation of dendritic cells (DCs) [36]. Reports have also demonstrated that PTX or docetaxel inhibited the NK cell-mediated killing of K562 target cells effectively in vitro [38, 39]. In the present study, the further optimization of the chemotherapy dosage regimen may bring results that are more reasonable. However, the decreased number of immune cells and the attenuation of the host immune system (Fig. 3) caused by PTX + CDDP treatment may have contributed to the uncontrolled tumor growth.

The anti-tumor mechanism of IL-12 is complex; previous studies have suggested that both innate and adaptive immunity are involved in the activity of IL-12 [17, 40, 41]. The anti-tumor effect of IL-12 on sarcomas or adenomas, which are considered immunogenic cancers [42], is mediated by CD4^+^ or CD8^+^ T cells [16]. In other tumor models, such as EL4, the IL-12-induced inhibition of tumor growth was shown to depend on NK and NKT cells [41]. It seems that the anti-tumor immune responses induced by IL-12 can be divided into two categories: adaptive immunity mediated primarily via IL-12-induced T_H_1 polarization, in which T cells are the primary effector cells, and innate immunity mediated via IL-12-activated NK or other innate immune cells. In one tumor model, the category of the cellular response induced by IL-12 may depend on tumor immunogenicity. In this report, we further revealed that NK cells play a major role in the anti-tumor effects of IL-12 on lung cancer (Fig. 4a, b). The inoculation of LLC cells induced a greater amount of tumor growth in NK cell-deficient mice (Nifl3^−/−^) than in WT mice. Moreover, in contrast to WT mice, the tumor growth in NK cell-deficient mice was not suppressed after IL-12 treatment (Fig. 4).

Given the importance of IFN-γ for the anti-tumor effects mediated by IL-12 [40], we used IFN-γ^−/−^ mice to further confirm the importance of this cytokine (Fig. 5). The major source of IFN-γ had not yet been elucidated, as both NK and T cells produce IFN-γ after IL-12 administration [18, 43]. Here, we revealed that NK cells can be activated rapidly and that intracellular IFN-γ was detected in these cells six hours after IL-12 administration. Notably, T cells could not be activated as quickly (Fig. 3b, e). IFN-γ and a cascade of other secondary and tertiary pro-inflammatory cytokines were reported to have a direct toxic effect on the tumor cells and cause tumor cell apoptosis [10]. Our results may suggest that IFN-γ may delay the onset of vigorous tumor growth. IFN-γ has also been reported to promote the inhibition of angiogenesis [44]. Subsequent experiments confirmed that CD31^+^ vessels in lung tumor tissue become expanded and disordered, particularly in the IFN-γ^−/−^ mice (Fig. 6). Vascular endothelial growth factor receptor 3 (VEGFR3), which is downregulated in quiescent adult vessels, is upregulated during angiogenesis [45, 46]. Moreover, VEGFR3 was abundantly expressed in lung tumor tissues, particularly in the IFN-γ^−/−^ mice. Importantly, the areas showing VEGFR3 expression did not overlap with CD31^+^ vessels in lung tumor tissue, suggesting the formation of new vessels. Further studies showed that CD31 and VEGFR3 expression was greatly reduced after IL-12 treatment and that weakly expressed VEGFR3 co-localized with CD31. IFN-γ may also induce the secretion of the chemokines IFN-γ-inducible protein 10 (IP-10) and monokine induced by IFN-γ (MIG) [47, 48]. These chemokines may induce alterations in the extracellular matrix remodeling process and decrease the expression of adhesion molecules from the endothelial cells [11, 49]. These mechanisms may lead to the IL-12-mediated anti-angiogenic effect of IFN-γ.

Toxic side effects are an important factor, which determines a drug’s clinical application. The Phase I and Phase II trials of IL-12 in patients with renal cancer were performed by the Genetics Institute. In the Phase I trial, the toxicity of IL-12 was acceptable. However, in the Phase II trial, the administration of IL-12 resulted in severe systemic toxicity that threatened patients’ lives [50, 51]. Subsequent investigations have found that the problem is triggered by the dosing regimen. In the Phase II study, the patients received consecutive high doses of IL-12 by intravenous injection. However, the Phase I trial gave patients various amounts of IL-12 to determine a maximum safe dose, which formed an “initial injection” of IL-12. Further studies in mice and cynomolgus monkeys revealed that an initial single dose of IL-12 prevented severe toxicity [50, 52]. In addition, the route of administration may impact the toxicity of IL-12. In another two Phase I trials, the toxicities of IL-12 from subcutaneous injection appeared to be mild and consisted mainly of a flu-like syndrome [53, 54]. Subsequent clinical trials using lower doses of IL-12 with subcutaneous injection have shown no severe systemic toxicity [20, 55]. Taken together, through the careful design of dosing regimen and administration method, the side effects of IL-12 can be minimized and acceptable.

## Conclusions

In summary, these results demonstrate that IL-12, either alone or in combination with PTX + CDDP, mediated significant anti-tumor activity and showed more efficacy than did PTX + CDDP alone in these lung cancer models. This mechanism is dependent on the rapid production of IFN-γ in activated NK cells, resulting in the inhibition of tumor angiogenesis. Hence, these findings may provide the basis for combination therapies to treat lung cancers, making this disease more accessible for targeted immune therapy. Although IL-12 has some side effects, through careful design of the dosing regimen and administration method, side effects can be minimized to acceptable levels, thus demonstrating that IL-12 is still a candidate for anticancer drugs.

## Additional files

Additional file 1: Figure S1. Analysis of lung tumor nodes and tumor lung tissues weight in the four treatment groups. (A) The LLC tumor nodes and lung tissues were photographed. The visible tumor nodes were counted, and the final lung tumor tissues were photographed after completion of the treatments. The tumor and lung tissues as a whole were weighed. The average visible lung tumor nodes and the weight of LLC tumor and lung tissues from the IL-12 or PTX + CDDP + IL-12 group were significantly less than those of the PTX or control group (n = 3; means ± SEM, ***P* < 0.01). (B) The CT26 tumor nodes and lung tissues were photographed. The visible tumor nodes were counted, and the final lung tumor tissues were photographed after completion of the treatments. The tumor and lung tissues as a whole were weighed. The average visible lung tumor nodes and the weight of CT26 tumor and lung tissues from the IL-12 or PTX + CDDP + IL-12 group were significantly less than those of the PTX or control group (*n* = 3; means ± SEM, ***P* < 0.01). (PDF 61 kb) Additional file 2: Figure S2. PTX + CDDP induce LLC cell apoptosis in vitro but not in vivo at the same concentration. (A) LLC cells were examined by micro-imaging and Tunel assay. PTX + CDDP induced LLC cell apoptosis in vitro (original magnification, ×200; scale bar, 50 μm). (B) Cryosections of tumor tissues were examined by Tunel. No apoptosis was detected in vivo in PTX + CDDP groups (original magnification, ×100; scale bar, 100 μm). (PDF 129 kb) Additional file 3: Figure S3. Spleens were enlarged after IL-12 treatment. (A) The spleen-size was calculated. The spleen-sizes increased significantly after IL-12 or PTX + CDDP + IL-12 treatment and were slightly reduced after PTX + CDDP treatment compared to the PTX + CDDP or the control groups (*n* = 3; means ± SEM, ***P* < 0.01). (B) The spleens were weighed. The weights of spleens increased significantly after IL-12 or PTX + CDDP + IL-12 treatment compared to the PTX + CDDP or control groups (*n* = 3; means ± SEM, **P* < 0.05). (PDF 36 kb) Additional file 4: Figure S4. Macrophage and T cell infiltration of tumors in the four treatment groups. Large number of F4/80^+^ macrophages infiltrated the tumor tissues in the four groups (original magnification, ×100; scale bar, 100 μm). Minimal infiltration of CD3^+^ T cells in tumor tissues in the four groups (original magnification, ×100; scale bar, 100 μm). (PDF 159 kb) Additional file 5: Figure S5. IL-12 treatment did not result in more invasive phenotype. (A) Matrix metalloprotein-9 (MMP-9) transcript level in para-carcinoma tissue was measured by quantitative PCR. MMP-9 transcript level was slightly decreased after IL-12 treatment (*n* = 3; means ± SEM, **P* < 0.05). (B) Cadherin 1 (CDH1) transcript level in para-carcinoma tissue was measured by quantitative PCR. Similar CDH1 transcript levels were found among the four groups. (PDF 5 kb)

## Abbreviations

BSA: Bovine serum albumin
CD: Cluster of differentiation
CDDP: Cisplatin
ELISA: Enzyme linked immunosorbent assay
FACS: Flow cytometric
H&E: Hematoxylin and eosin
IFN-γ: Interferon-γ
IL: Interleukin
IP-10: IFN-γ-inducible protein 10
LLC: Lewis lung carcinoma
MHC: Major histocompatibility complex
MIG: Monokine induced by IFN-γ
Nfil3: Nuclear factor interleukin-3
NKSF: Natural killer cell stimulatory factor
NS: Normal saline
NSCLC: Non-small cell lung cancer
PTX: Paclitaxel
SCLC: Small cell lung cancer
SD: Stable disease
Th: T-helper
VEGFR3: Vascular endothelial growth factor receptor 3
VEGFR3: vascular endothelial growth factor receptor 3
WT: Wild type
