# Supplementary figures and images for: Interleukin 12 shows a better curative effect on lung cancer than paclitaxel and cisplatin doublet chemotherapy
Source: BMC Cancer. 2016 Aug 22;16(1):665. doi: 10.1186/s12885-016-2701-7 (PMC4994391; doi:10.1186/s12885-016-2701-7)

**A**

**LLC**

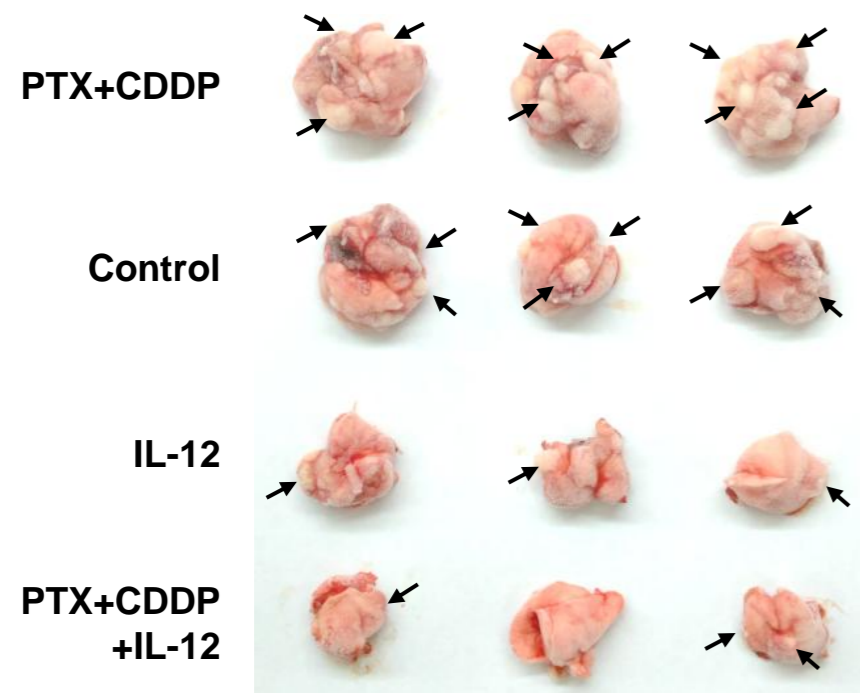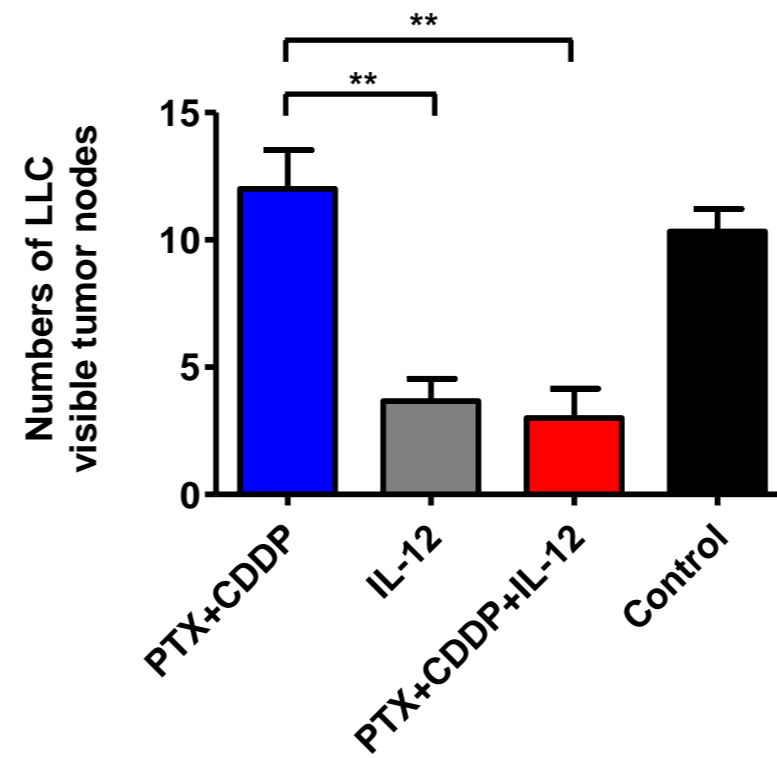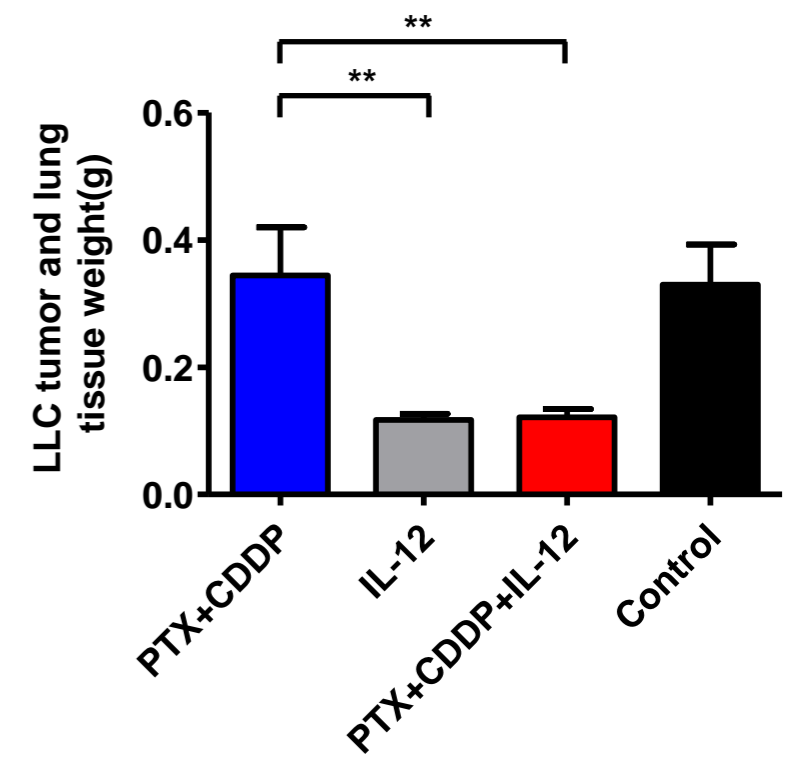

**B**

**CT26**

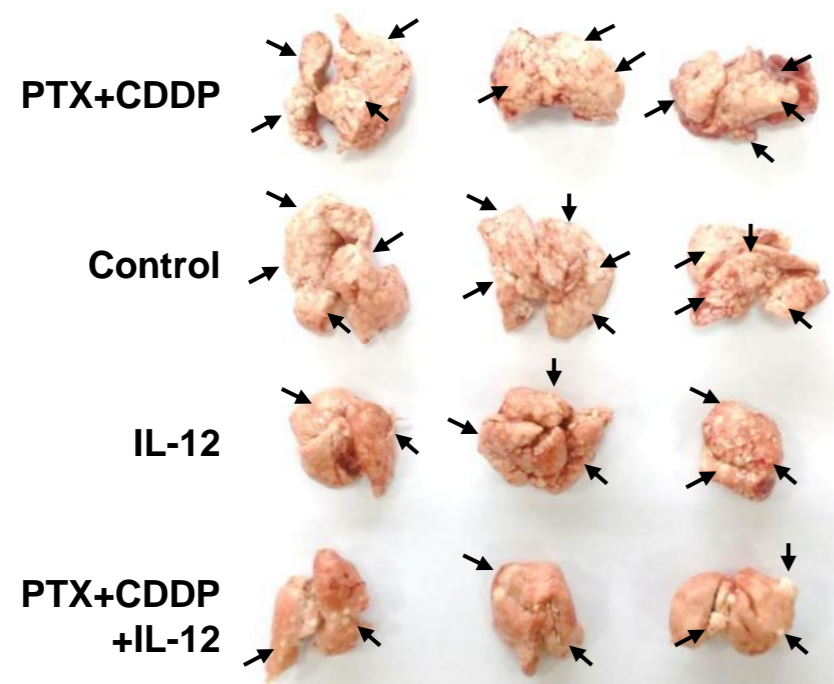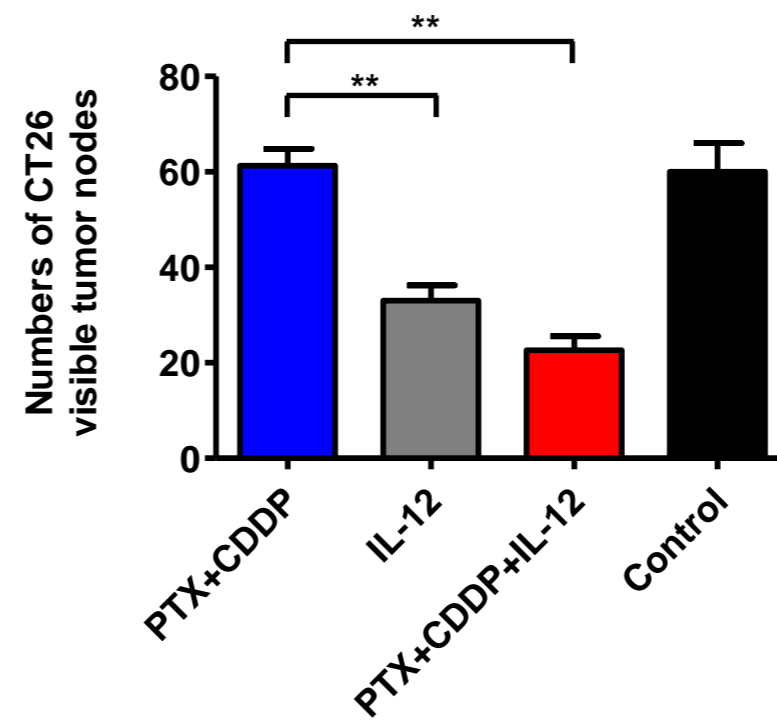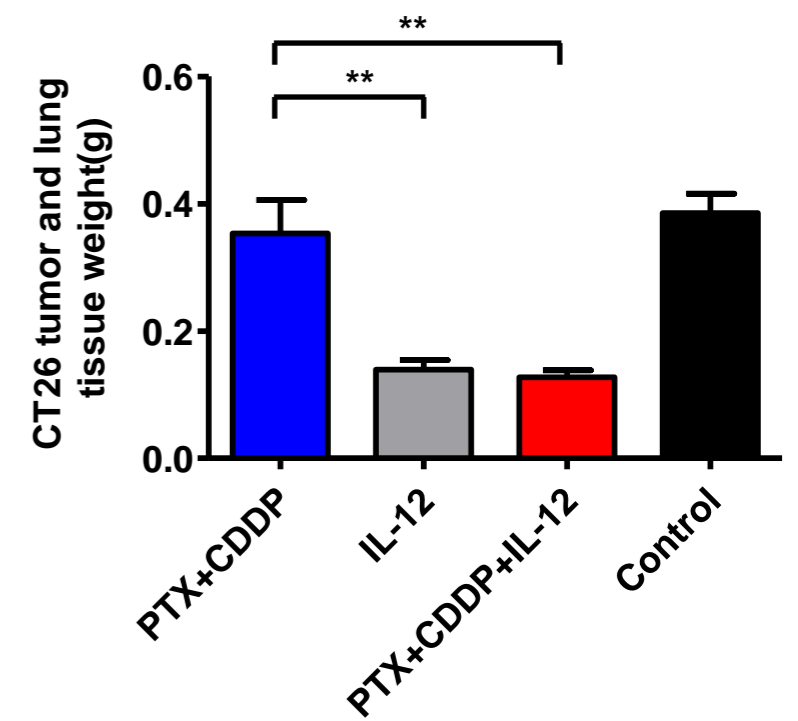

Supplement: Additional file 1: Figure S1. — Analysis of lung tumor nodes and tumor lung tissues weight in the four treatment groups. (A) The LLC tumor nodes and lung tissues were photographed. The visible tumor nodes were counted, and the final lung tumor tissues were photographed after completion of the treatments. The tumor and lung tissues as a whole were weighed. The average visible lung tumor nodes and the weight of LLC tumor and lung tissues from the IL-12 or PTX + CDDP + IL-12 group were significantly less than those of the PTX or control group (n = 3; means ± SEM, **P < 0.01). (B) The CT26 tumor nodes and lung tissues were photographed. The visible tumor nodes were counted, and the final lung tumor tissues were photographed after completion of the treatments. The tumor and lung tissues as a whole were weighed. The average visible lung tumor nodes and the weight of CT26 tumor and lung tissues from the IL-12 or PTX + CDDP + IL-12 group were significantly less than those of the PTX or control group (n = 3; means ± SEM, **P < 0.01). (PDF 61 kb) [file 12885_2016_2701_MOESM1_ESM.pdf]

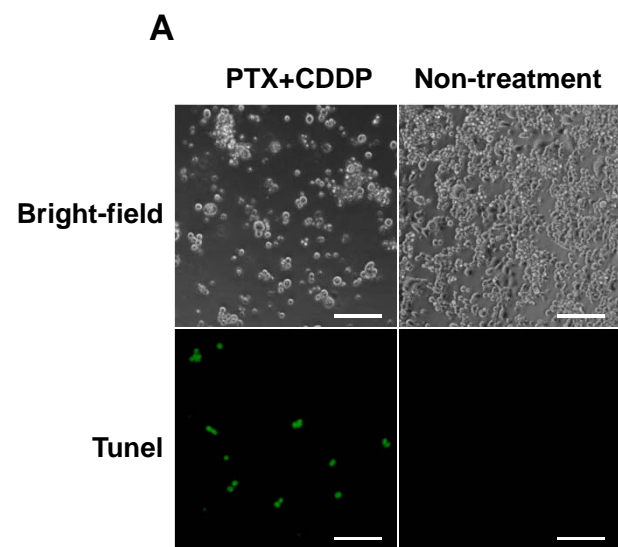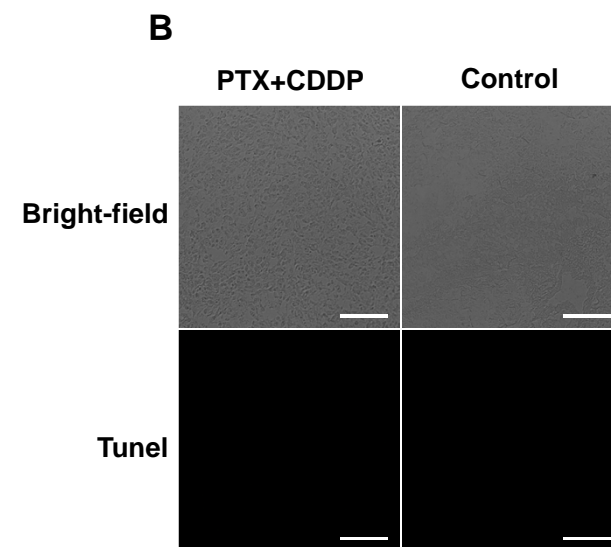

Supplement: Additional file 2: Figure S2. — PTX + CDDP induce LLC cell apoptosis in vitro but not in vivo at the same concentration. (A) LLC cells were examined by micro-imaging and Tunel assay. PTX + CDDP induced LLC cell apoptosis in vitro (original magnification, ×200; scale bar, 50 μm). (B) Cryosections of tumor tissues were examined by Tunel. No apoptosis was detected in vivo in PTX + CDDP groups (original magnification, ×100; scale bar, 100 μm). (PDF 129 kb) [file 12885_2016_2701_MOESM2_ESM.pdf]

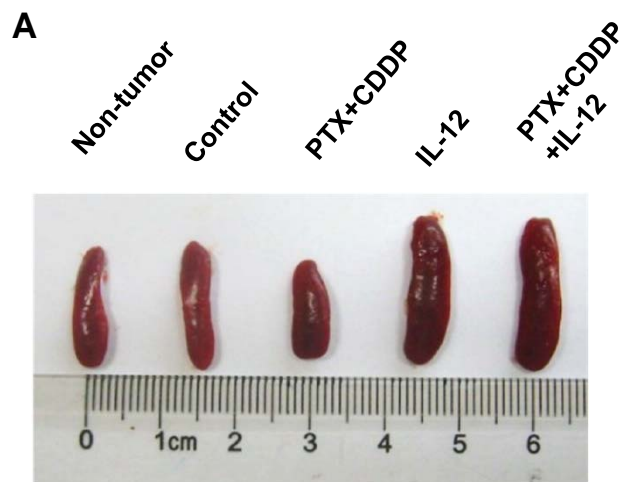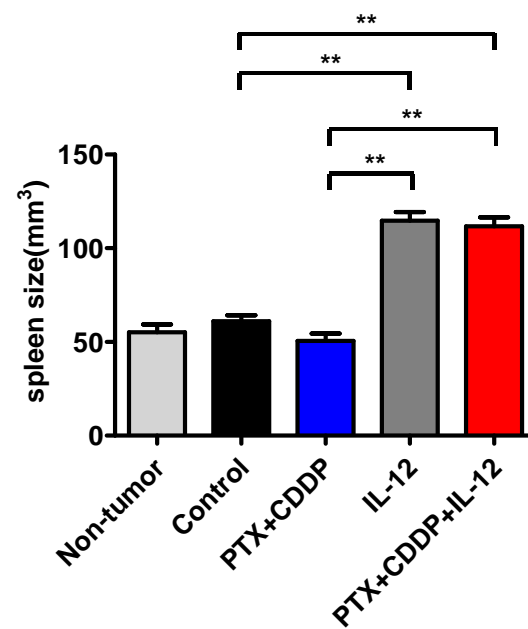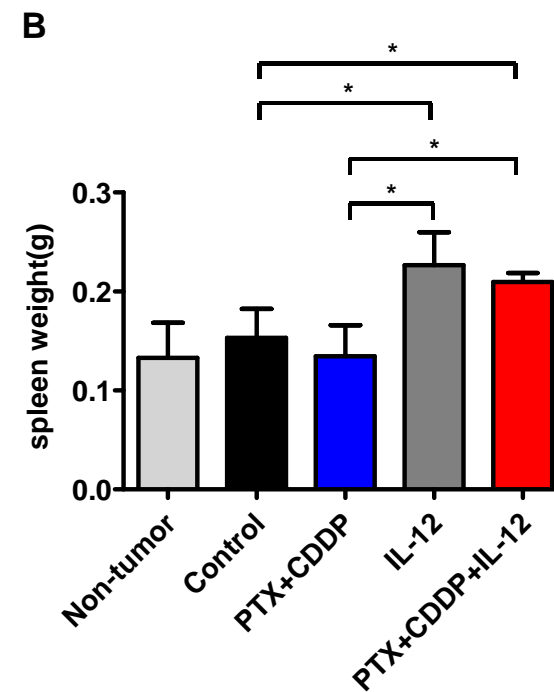

Supplement: Additional file 3: Figure S3. — Spleens were enlarged after IL-12 treatment. (A) The spleen-size was calculated. The spleen-sizes increased significantly after IL-12 or PTX + CDDP + IL-12 treatment and were slightly reduced after PTX + CDDP treatment compared to the PTX + CDDP or the control groups (n = 3; means ± SEM, **P < 0.01). (B) The spleens were weighed. The weights of spleens increased significantly after IL-12 or PTX + CDDP + IL-12 treatment compared to the PTX + CDDP or control groups (n = 3; means ± SEM, *P < 0.05). (PDF 36 kb) [file 12885_2016_2701_MOESM3_ESM.pdf]

DAPI    CD3    F4/80

PTX+CDDP

IL-12

PTX+CDDP+IL-12

Control

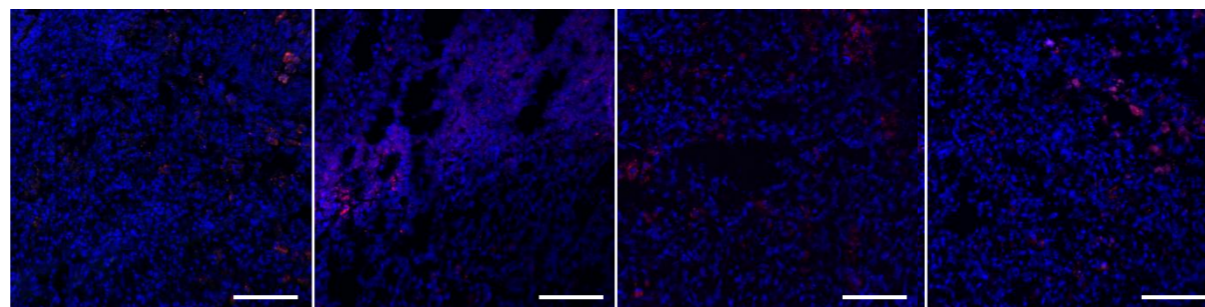

PTX+CDDP

IL-12

PTX+CDDP+IL-12

Control

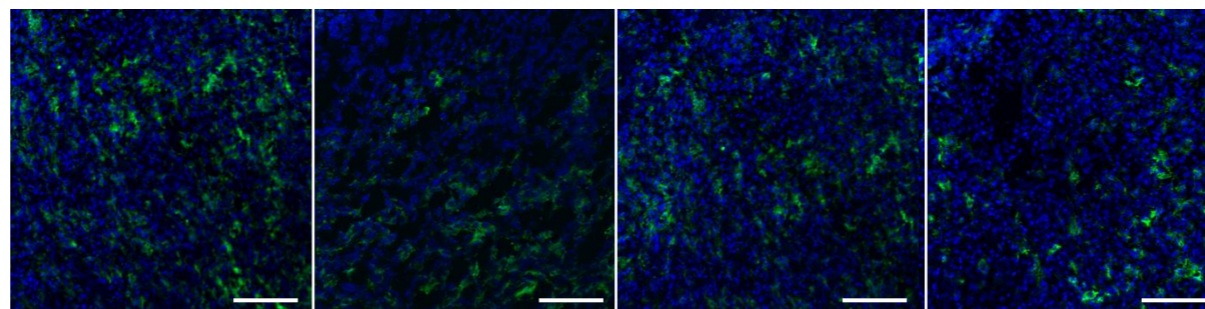

Supplement: Additional file 4: Figure S4. — Macrophage and T cell infiltration of tumors in the four treatment groups. Large number of F4/80+ macrophages infiltrated the tumor tissues in the four groups (original magnification, ×100; scale bar, 100 μm). Minimal infiltration of CD3+ T cells in tumor tissues in the four groups (original magnification, ×100; scale bar, 100 μm). (PDF 159 kb) [file 12885_2016_2701_MOESM4_ESM.pdf]

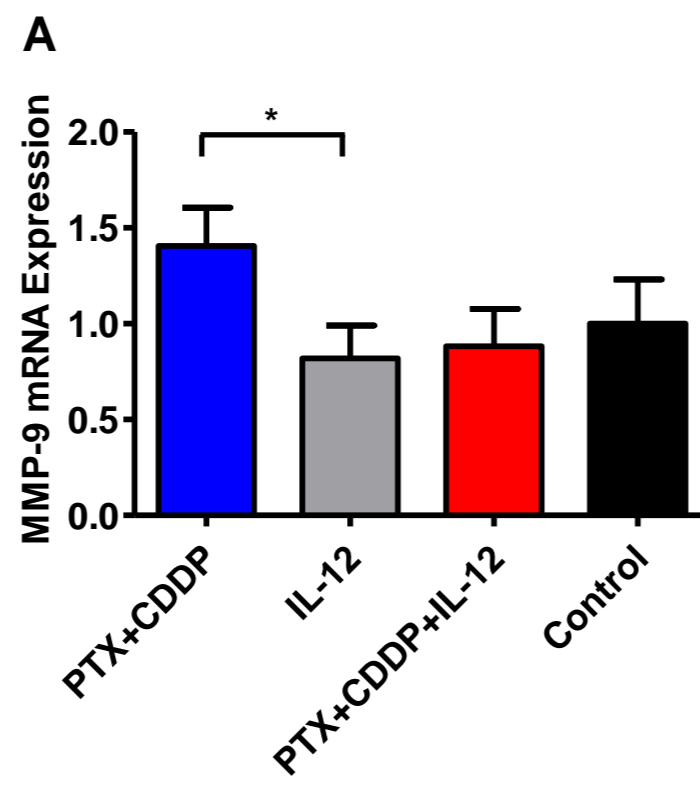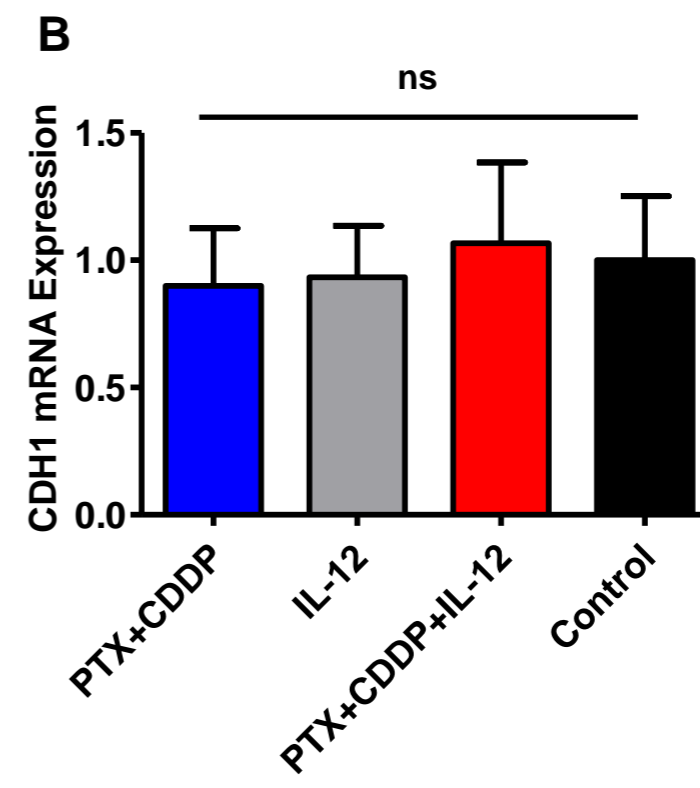

Supplement: Additional file 5: Figure S5. — IL-12 treatment did not result in more invasive phenotype. (A) Matrix metalloprotein-9 (MMP-9) transcript level in para-carcinoma tissue was measured by quantitative PCR. MMP-9 transcript level was slightly decreased after IL-12 treatment (n = 3; means ± SEM, *P < 0.05). (B) Cadherin 1 (CDH1) transcript level in para-carcinoma tissue was measured by quantitative PCR. Similar CDH1 transcript levels were found among the four groups. (PDF 5 kb) [file 12885_2016_2701_MOESM5_ESM.pdf]
